# Oviposition response of *Aedes* mosquitoes to different cement types: a field-based study in urban Sri Lanka

**DOI:** 10.1186/s13104-025-07610-8

**Published:** 2025-12-13

**Authors:** Yasodha Ranasinghe, Nayana Gunathilaka, Deshaka Jayakody, Wasana Rodrigo

**Affiliations:** 1 Department of Zoology, Faculty of Natural Sciences, Open University of Sri Lanka, Nugegoda, Sri Lanka; 2 Department of Parasitology, Faculty of Medicine, University of Kelaniya, Ragama, Sri Lanka

**Keywords:** Dengue, Vectors, Oviposition, Cement types

## Abstract

**Background:**

Dengue is a rapidly expanding vector-borne disease transmitted primarily by *Aedes* mosquitoes, which often breed in artificial containers. Urban construction sites, with their abundance of water-holding surfaces, are increasingly recognized as key breeding habitats. This study presents the first field-based evaluations of how specific construction materials, particularly cement types, influence mosquito oviposition behavior.

**Method:**

A seven-month field-based, randomized controlled experiment (September 2024–March 2025) was conducted across three Grama Niladhari divisions (Kolonnawa, Salamulla, and Orugodawatta) in Colombo District, Sri Lanka. A total of 480 ovitraps using four substrate types, Control (plastic), Blended Hydraulic Cement (BHC), Ordinary Portland Cement (OPC), and Portland Limestone Cement (PCC), were deployed in indoor (*n* = 4) and outdoor (*n* = 4) settings at 20 randomly selected households per GN division. Water quality parameters (pH, turbidity, conductivity) were recorded at trap placement. Egg counts were analyzed using a Zero-Inflated Negative Binomial (ZINB) regression model.

**Results:**

Traps containing BHC and PCC substrates had significantly fewer eggs than controls (BHC: β = -0.402, *P* = 0.014; PCC: β = -0.527, *P* = 0.001) and showed a higher likelihood of zero-egg presence (BHC: β = 1.025, *P* = 0.002; PCC: β = 0.941, *P* = 0.003). Outdoor traps had higher egg counts than indoor ones (β = 0.326, *P* = 0.021). Among water quality factors, only conductivity was significantly associated with egg counts (*P* = 0.036).

**Conclusion:**

The BHC and PCC cement types significantly deterred *Aedes* oviposition compared to other substrates, indicating that construction materials can influence mosquito breeding behavior.

**Supplementary Information:**

The online version contains supplementary material available at 10.1186/s13104-025-07610-8.

## Background

Dengue is the most prevalent viral infection in tropical and sub-tropical countries, infecting between 100 and 400 million people per year [1] in over 125 countries, resulting in 10,000 deaths per year [2]. In Sri Lanka, dengue has become a persistent public health challenge, with frequent outbreaks and a rising number of cases reported each year, particularly from urban and peri-urban areas such as Colombo District. *Aedes aegypti* and *Aedes albopictus* are the primary and secondary vectors, respectively, for dengue transmission in Sri Lanka [3, 4].

*Aedes* mosquitoes exhibit diverse oviposition behaviors influenced by various factors. They prefer containers with easy access and dark surfaces as initial attractants [5]. Volatile chemicals from decaying vegetation serve as close-range attractants, while water quality and food availability determine egg deposition [5]. Oviposition site selection is crucial for egg and larval survival, especially in short-lived aquatic habitats [6]. Different mosquito species show preferences for specific container types and sizes, with large containers generally having the highest mosquito densities and species richness [7]. *Aedes aegypti*, in particular, prefers to oviposit close to the ground in open water containers with organic compounds from plant watering [8]. Visual and chemical cues, such as potted plant shape and organic matter odor, are equally attractive to gravid females [8]. Studies have shown that *Ae. aegypti* and *Ae. albopictus* prefer to lay eggs in organic infusions and larval-holding water over plain water [9, 10]. Specific bacteria-associated compounds, including carboxylic acids and methyl esters, act as potent oviposition stimulants for *Ae. aegypti* [11]. Certain household substances, such as salt, vinegar, and some plant extracts, can deter oviposition, while others like cumin seeds may attract egg-laying [12]. These findings suggest that *Aedes* mosquitoes can detect chemical differences in water sources, which significantly influences their choice of oviposition sites. When considering *Ae. aegypti*, their ability to transition from natural breeding sites like tree holes to artificial containers in human settlements has been crucial to their success as disease vectors [13]. This flexibility in oviposition site selection allows them to utilize human-stored water during dry seasons, potentially initiating their domestic evolution [13].

Rapidly increasing rate of urbanization has provided the *Aedes* mosquito with many new breeding sites, and this increase in breeding sites has become an important risk factor for dengue fever [14]. Urbanization is changing the natural environment, transforming rural areas into urban areas and expanding population distribution in urban areas from rural areas [15]. Construction sites are an integral part of the urbanization process. As construction sites are dynamic environments, different construction phases may allow the sites to be conducive for *Aedes* breeding when potential habitats are constantly created or not removed. For example, water puddles on various surfaces, such as concrete floors, are in uncompleted building habitats for *Ae. aegypti* [16]. On the other hand, construction sites are with lack of basic mosquito control facilities. Therefore, construction sites have made high-risk areas hotspots for outbreaks of dengue fever [14].

The chemical composition of cementitious materials can appreciably influence the physicochemical properties of standing water in contact with them [17, 18]. Hydration reactions and leaching from cement can alter parameters such as pH, conductivity, salinity, and turbidity [19]. These shifts in water quality may, in turn, affect mosquito oviposition behaviors and egg viability [20], making them ecologically relevant endpoints, because the development of immature stages depends on the water characteristics of the container, including nutrient content, water volume, pH, temperature, conductivity, and dissolved oxygen, among others [21]. The main chemical composition of the cement in terms of SiO_2_, Al_2_O_3_, Fe_2_O_3_, Fe_2_O_3_, CaO, MgO, and SO_3,_ and the percentage of that compound broadly varies in cement types [22]. There are several types of cement available in the Sri Lanka market, namely Ordinary Portland Cement (OPC), Blended Hydraulic Cement (BHC), Portland Limestone Cement (PLC), Portland Composite Cement (PCC) and Masonry Cement (MC). Therefore, water characteristics of the breeding places at construction sites may vary in different construction sites. The chemical composition of the major cement materials used in Sri Lanka is provided in the supplementary material (Table S1) [23].

Furthermore, there are different cement brands in the Sri Lankan market. However, the percentage of chemical composition is narrow among the chosen cement types of different cement brands [24]. With the rapid expansion of urban areas, cement-based constructions have become increasingly common, and there is growing interest in how these materials might influence the oviposition behaviour of dengue vectors. Understanding whether cement surfaces or structures attract mosquitoes could provide valuable insights for vector control and urban planning strategies.

This is the first field-based study in Sri Lanka to assess how commonly used cement materials influence *Aedes* mosquito oviposition, addressing a critical gap in construction-related vector ecology. This study aims to investigate how cement types used in construction affect *Aedes* oviposition.

## Methods

### Study area

This study was conducted in the Kolonnawa Medical Officer of Health (MOH) area, located in the Colombo District of Sri Lanka, an urban region characterized by rapid urbanization and a high incidence of dengue, with 238 cases reported in 2023 and 149 cases in 2024. Three Grama Niladhari (GN) divisions, namely, Kolonnawa, Salamulla, and Orugodawatta were selected based on prior entomological surveillance data, which reported higher Breteau Index (BI) values, indicating elevated levels of *Aedes* mosquito breeding activity in these areas. Kolonnawa, Salamulla, and Orugodawatta, being neighbouring areas within Colombo District, Sri Lanka, share a similar tropical climate. The average annual temperature is around 30 °C, with highs near 32 °C and lows about 27 °C. The average relative humidity is roughly 83%, reflecting a persistently humid environment. These areas receive substantial rainfall throughout the year, with an annual average precipitation close to 345 mm, and nearly 80% of days experiencing some rain [25].

### Study design and selection of study sites

**Fig. 1:**
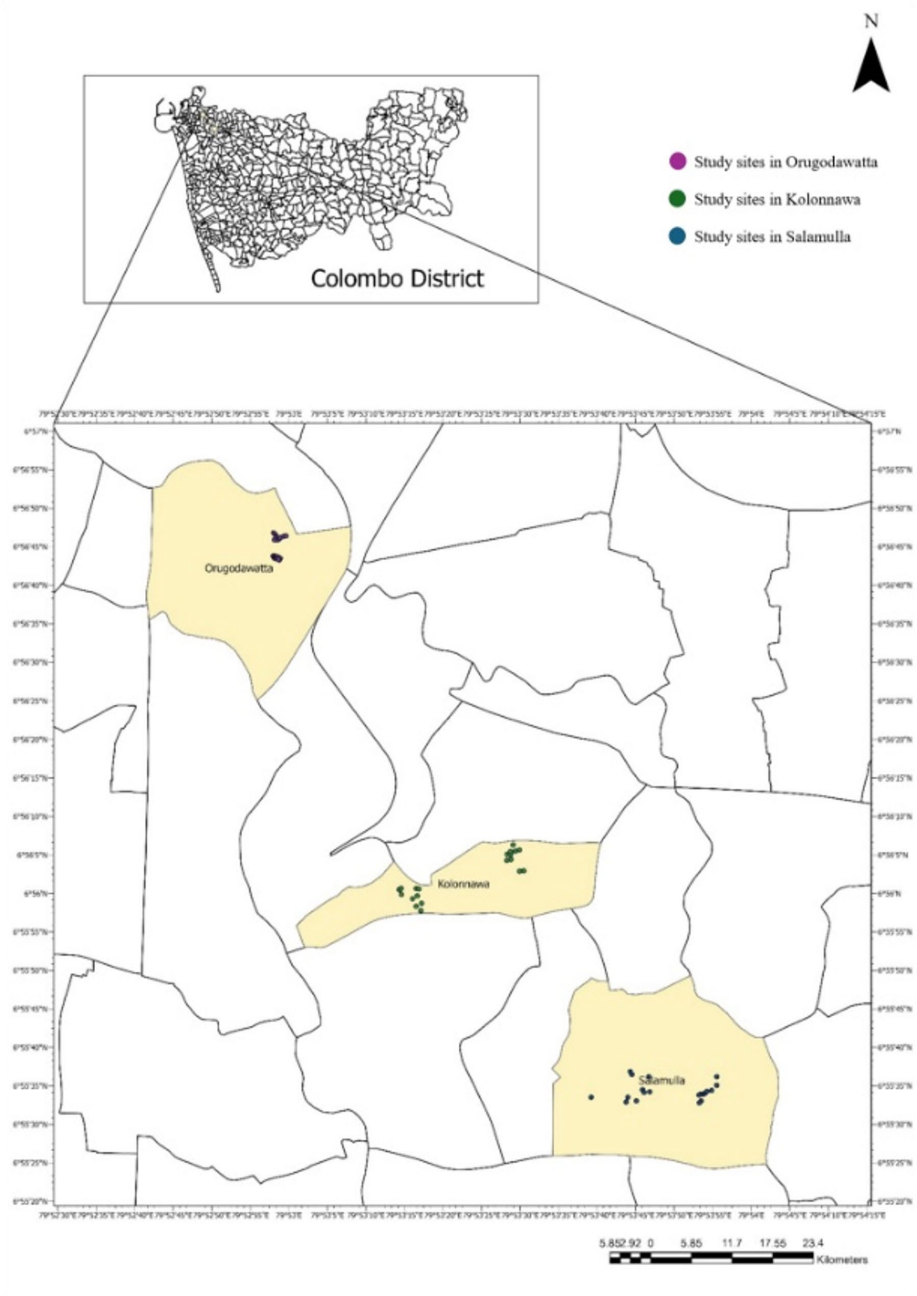
Map of the study area within the Kolonnawa Medical Officer of Health (MOH) division, Colombo District, Sri Lanka. The three Grama Niladhari (GN) divisions where the study was conducted Kolonnawa, Salamulla, and Orugodawatta are highlighted. The locations of the selected households and study sites are also indicated

This study employed a field-based, randomized controlled experimental design with repeated measures to evaluate the oviposition preferences of dengue vectors (*Aedes* mosquitoes) in relation to different cement types (OPC, BHC, PCC) commonly used in construction for a period of 7 months from September 2024 to March 2025. A total of 60 premises were randomly selected for the study, with 20 premises from each of the three GN divisions (Fig. 1). Properties located in these three GN divisions, primarily residential in nature, were used as the inclusion criteria. Premises that are undergoing construction or major renovations, a history of regular vector control measures, and presently under a control intervention, premises on which the owners may not provide consent to set traps, were not considered in selecting the site for ovitrap placement.

## Ovitrap preparation and standardization

**Fig. 2:**
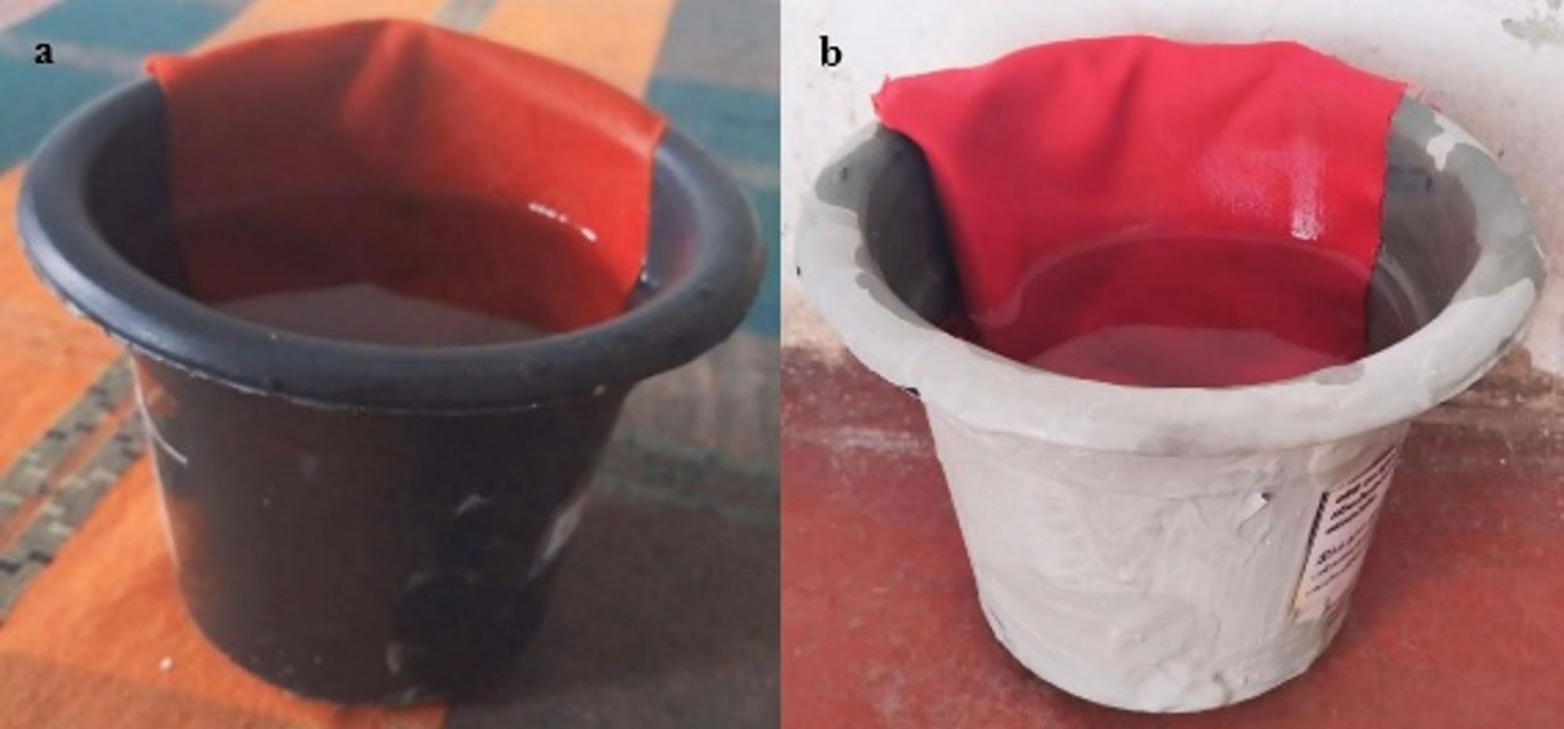
Ovitraps used in the study. **a** Standard control ovitrap consisting of a black plastic container filled with water and lined with a red cotton cloth strip. **b** Experimental ovitrap constructed from cement, similarly filled with water and lined with a red cotton cloth strip

Four types of ovitraps were used in the study: a control trap and three experimental traps made with different cement types: Ordinary Portland Cement [OPC], Blended Hydraulic Cement [BHC], and Portland Limestone Cement [PCC]. The control ovitrap was a standard black plastic container with a volume of 300 mL. The cement-based ovitraps were moulded to match the size and shape of the control trap using a cement-to-water ratio of 3:1. After moulding, all cement ovitraps were cured and then stored in a well-ventilated, mosquito-free area for 7 days to allow any residual odors or volatile compounds to dissipate before deployment in the field. A standardized volume of 250 mL of water, sourced from the same location was added to each ovitrap. A red cotton cloth strip was used as the ovistrip in all traps, placed along the inner wall so that it was half-submerged in water and half exposed above the surface, providing an oviposition substrate (Fig. 2).

## Ovitap survey

A total of 480 ovitraps were used for the study; Control without cement material, BHC, OPC, and PCC) were placed in indoor (*n* = 4) and outdoor (*n* = 4) settings (one with each substrate) at 20 randomly selected households in each GN division. Ovitraps were placed at a minimum distance of 10 cm from each other. Outdoor ovitraps were positioned in shady areas to provide suitable conditions for oviposition and were placed in locations free from disturbances. The ovitraps were collected after the 4th day of the initial setup for oviposition screening [26].

### Water quality monitoring

Water quality monitoring was conducted during the placement of the ovitraps to assess the impact of various parameters on mosquito oviposition behavior. Upon placing each ovitrap, the physicochemical parameters of the water in the ovitrap were tested using a portable detector including pH, conductivity, and turbidity.

### Collection of eggs from ovitraps and egg counting

Eggs were collected from ovitraps four days after placement to allow sufficient time for *Aedes* mosquitoes to deposit eggs. Each ovitrap was carefully retrieved while wearing disposable gloves to prevent contamination, and the entire contents, including water and ovistrips were placed in labelled containers for transportation to the laboratory. In the laboratory, the ovitrap contents were poured into petri dishes for examination. Any adhering eggs were collected by rinsing the ovitraps with distilled water. A dissecting microscope was used to accurately count the eggs, which were recorded in a data collection system, including details such as premise ID, ovitrap type, and total egg count. To account for eggs that had hatched before harvesting, counting included both intact and hatched eggs; even when larvae had emerged, the opened eggshells remained visible on the ovistrips, allowing an accurate estimate of total eggs laid. After counting, the eggs and water were placed back into clean water to allow for further hatching, and the number of larvae was recorded and identified at the species level using a dichotomous key [27]. Quality control involved random recounting by a second researcher to ensure accuracy.

### Data analysis

Due to the high proportion of zero values in the egg count data (62.70%), a Zero-Inflated Negative Binomial (ZINB) regression model was selected to account for the excess zeros. The variable salinity was excluded from the model because it exhibited no variation across all traps, thereby providing no explanatory power. The final model included the following explanatory variables: oviposition substrate (Control, BHC, OPC, and PCC), with the Control substrate serving as the reference category for comparison with the effects of different cement types; trap location (Indoor vs. Outdoor), with Indoor as the reference category; GN Division (Kolonnawa, Orugodawatta, and Salamulla), with Kolonnawa as the baseline category; and water quality parameters (pH, conductivity, and turbidity), measured prior to oviposition. The results of the ZINB model are presented in two parts: the count model (negative binomial component), which estimates egg counts, and the zero-inflation model (logit component), which estimates the probability of a trap yielding zero eggs.

The goodness of fit of the ZINB model was evaluated using several diagnostic methods. To assess predictive performance, a scatter plot of predicted versus observed egg counts was generated. A residual versus predicted plot was created to identify any patterns or potential heteroscedasticity. Additionally, a binned residual plot was used to check for systematic biases in prediction across the range of fitted values.

All analyses were conducted in R (version 4.4.2). The following R packages were used to perform data handling, model fitting, and visualization: readxl, pscl, MASS, car, ggplot2, arm, and dplyr.

## Results

### Effect of ovitrap substrate type on oviposition preference

**Fig. 3:**
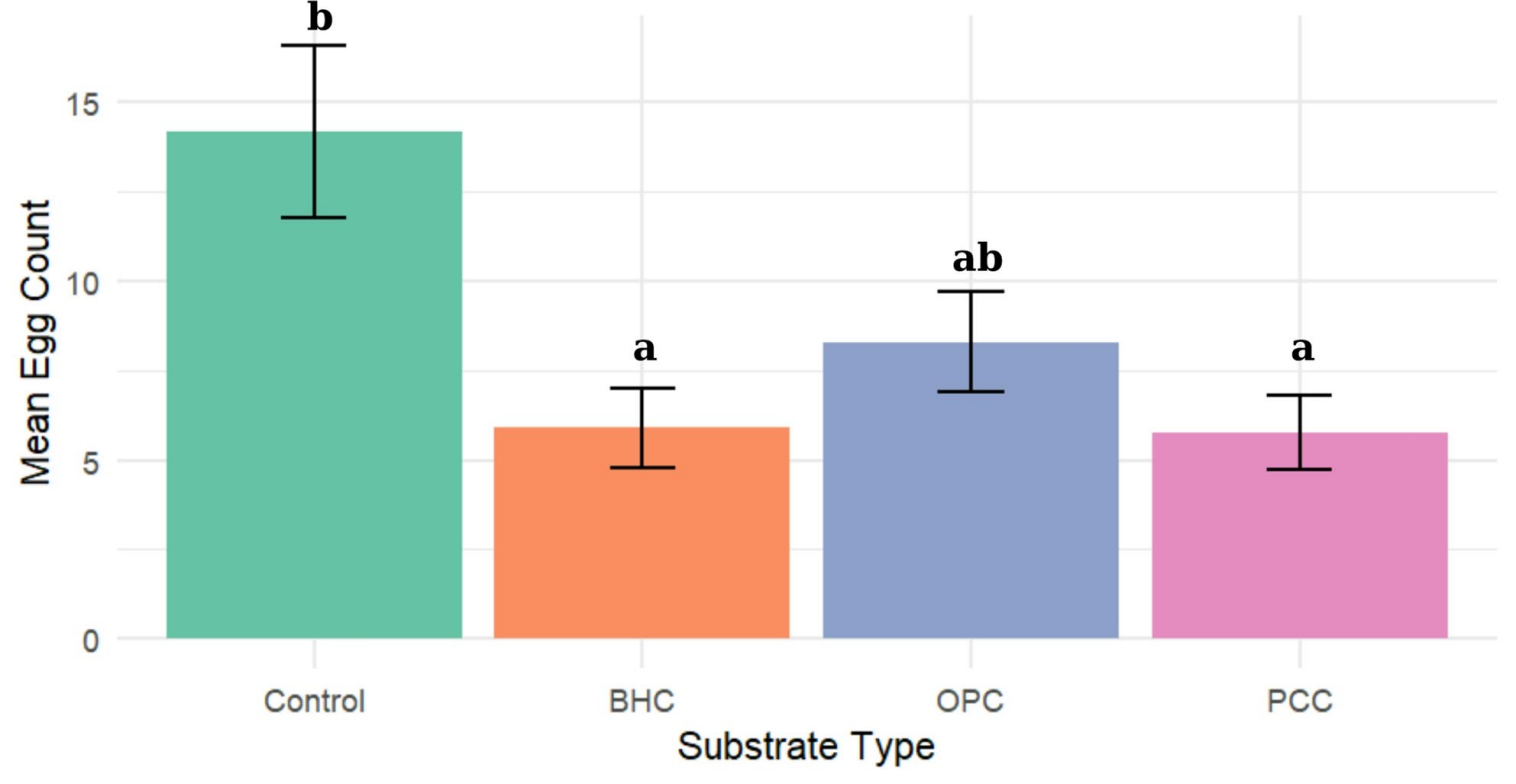
Mean egg count (± SE) observed across different substrate types. BHC = Blended Hydraulic Cement; OPC = Ordinary Portland Cement; PCC = Portland Limestone Cement. Different letters indicate statistically significant differences between groups based on Dunn’s post hoc test following a Kruskal-Wallis test (*P* < 0.05)

There was a notable difference in oviposition preference among the tested cement types. The control substrate recorded the highest mean egg count (14.15 ± 2.41), indicating its greater attractiveness to ovipositing females. In contrast, BHC and PCC substrates indicated considerably lower mean egg counts, suggesting reduced suitability or preference (Fig. 3). The OPC substrate denoted a moderate response, with a mean egg count of 8.28 ± 1,39, which was higher than BHC (5.90 ± 1.11) and PCC (5.78 ± 1.02), but still lower than the control.

### Comparison of oviposition activity between indoor and outdoor environments

**Fig. 4:**
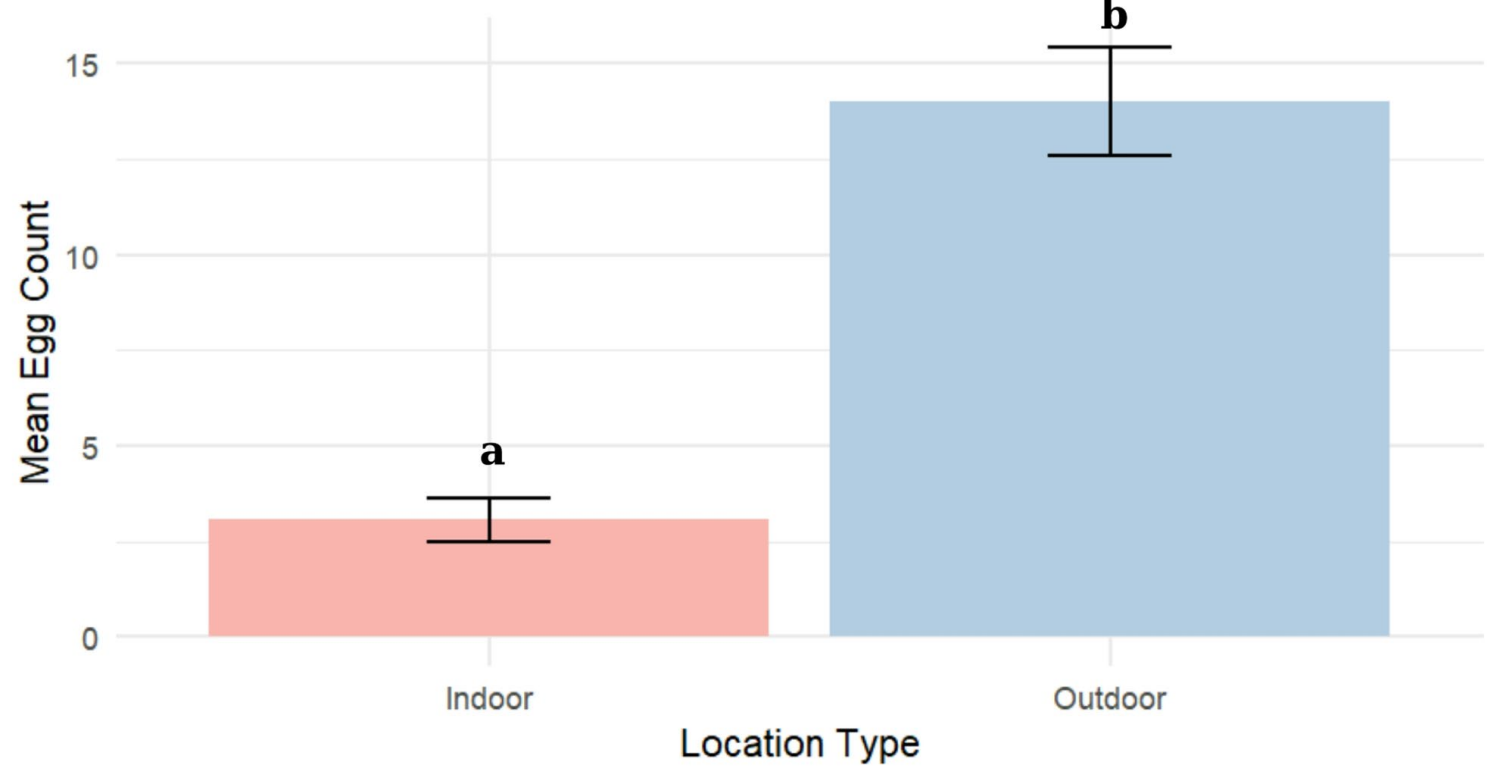
Mean egg count (± SE) in ovitraps placed indoors and outdoors. Different letters indicate statistically significant differences between locations based on the Mann–Whitney U test (*P* < 0.05)

There was a clear disparity in oviposition activity, with outdoor traps recording a higher mean egg count (14 ± 1.42) compared to indoor traps, which had a mean of 3.06 ± 0.56 eggs. This indicated a strong preference for outdoor environments by ovipositing females (Fig. 4).

### Determining the selection of cement types for oviposition

The Zero-Inflated Negative Binomial (ZINB) regression model identified several significant predictors of *Aedes* egg counts and the likelihood of observing zero-egg ovitraps. Conductivity was the only water quality parameter significantly associated with egg counts (*P* = 0.036), showing a negative effect, indicating that higher conductivity levels reduced *Aedes* oviposition. Among the oviposition substrates, BHC (*β* = -0.402; *P* = 0.014) and PCC (*β* = -0.527; *P* = 0.001) traps had significantly fewer eggs compared to the control substrate (Table 1). Outdoor traps yielded significantly higher egg counts than indoor traps (*β* = 0.326; *P* = 0.021). Among GN divisions, traps in Orugodawatta had significantly lower egg counts than those in Kolonnawa (*β* = -0.694; *P* < 0.001), while no significant difference was observed in Salamulla area (Table 1).

**Table 1:** Predictors of *Aedes* egg count in ovitraps based on cement type, water properties, and trap placement: Zero-inflated binomial regression

Predictor	Estimate	Std. Error	z-value	*P*-value
(Intercept)	3.102	0.662	4.685	< 0.001***
pH	0.032	0.059	0.538	0.591
Conductivity	-0.001	0.0007	-2.092	0.036*
Turbidity	0.0001	0.013	0.01	0.992
BHC (vs. Control)	-0.402	0.164	-2.445	0.014*
OPC (vs. Control)	-0.249	0.157	-1.584	0.113
PCC (vs. Control)	-0.527	0.161	-3.266	0.001**
Outdoor (vs. Indoor)	0.326	0.141	2.313	0.021*
Orugodawatta (vs. Kolonnawa)	-0.694	0.163	-4.268	< 0.001***
Salamulla (vs. Kolonnawa)	-0.027	0.142	-0.192	0.848

*significant at *P* < 0.05

**significant at *P* < 0.01

***significant at *P* < 0.001

Both BHC (*β* = 1.025; *P* = 0.002) and PCC (*β* = 0.941; *P* = 0.003) were associated with a significantly higher probability of traps being egg-negative. Outdoor traps were also more likely to be egg-negative compared to indoor traps (*β* = -1.994; *P* < 0.001). Additionally, traps located in Orugodawatta (*β* = 1.545, *p* < 0.001) and Salamulla (*β* = 1.027; *P* < 0.001) had a significantly higher likelihood of yielding zero eggs than those in Kolonnawa area (Table 2).

**Table 2:** Predictors of zero-egg counts in ovitraps by cement type, water quality, and trap placement: Zero-inflated binomial regression

Predictor	Estimate	Std. Error	z-value	*p*-value
(Intercept)	1.186	1.212	0.978	0.328
pH	-0.088	0.113	-0.777	0.437
Conductivity	~ 0	0.0015	-0.056	0.956
Turbidity	-0.023	0.03	-0.769	0.442
BHC (vs. Control)	1.025	0.325	3.157	0.002**
OPC (vs. Control)	0.589	0.319	1.847	0.065
PCC (vs. Control)	0.941	0.315	2.986	0.003**
Outdoor (vs. Indoor)	-1.994	0.235	-8.492	< 0.001***
Orugodawatta (vs. Kolonnawa)	1.545	0.309	5.008	< 0.001***
Salamulla (vs. Kolonnawa)	1.027	0.283	3.627	< 0.001***

*significant at *P* < 0.05

**significant at *P* < 0.01

***significant at *P* < 0.001

### Goodness of fit of the model

To evaluate the performance and adequacy of the Zero-Inflated Negative Binomial (ZINB) model in capturing the patterns of *Aedes* egg counts, several diagnostic plots were examined. The majority of the data points are closely aligned with the 45-degree reference line, indicating a strong agreement between the model’s predictions and the actual observations (Supplementary Figure S1a). This alignment suggests that the model has reasonable predictive accuracy and effectively captures the overall trend in the data. The residuals plotted against the predicted egg counts is illustrated in Supplementary Figure S1b. The residuals are symmetrically distributed around the zero line with no discernible patterns, heteroscedasticity, or funnel shapes. This indicates that the model errors are randomly distributed, supporting the assumption of homoscedasticity and suggesting that the model does not systematically under predict or over predict egg counts across the range of predicted values.

The binned residual plot, further confirms the model’s adequacy. In this plot, the residuals were grouped into bins based on predicted values, and the average residual in each bin was plotted with corresponding confidence intervals (Supplementary Figure S1c). Most binned residuals fell within the 95% confidence bands, with no clear deviations or systematic bias across the spectrum of fitted values. This suggests that the model’s assumptions are largely met and that the model is well-calibrated for the data at hand.

### Species composition in different substrates

The analysis of species composition across different cement types showed no significant differences among the groups. This was confirmed by the Kruskal-Wallis test, a non-parametric method employed due to the non-normal distribution of the data. The test results indicated no statistically significant differences in the larval counts of *Ae. aegypti* larval count (χ² = 1.2908, *P* = 0.731), or *Ae. albopictus* larval count (χ² = 1.2733, *P* = 0.736) among the cement types.

## Discussion

Urban vector ecology studies across South and Southeast Asia consistently show that *Ae. aegypti* and *Ae. albopictus* adapts well to urban environments due to the abundance of artificial containers and rapid urbanization, which create diverse oviposition sites [28, 29]. Multiple studies from Sri Lanka and the wider region confirm that *Aedes* species exhibit strong container preferences influenced by water chemistry, with plastic and other inert containers often supporting higher egg densities [30, 31], while elevated salinity or dissolved solids such as those leached from construction materials tend to deter oviposition [32]. Furthermore, skip-oviposition (eggs laid in multiple containers during a single cycle) is well documented as a key adaptive strategy in both *Ae. aegypti* and *Ae. albopictus*, increasing survival chances in variable urban habitats [33]. The ecological plasticity of these mosquitoes in the exploitative use of changing urban landscapes, including construction-driven habitats, corresponds with observed dengue risks in rapidly urbanizing regions [28, 29].

This study provides novel field-based evidence on how different cement-based materials used in construction sites influence the oviposition behavior of *Aedes* mosquitoes, particularly *Ae. aegypti* and *Ae. albopictus*. The results highlight the significant role that construction-related substrates play in vector ecology, especially in rapidly urbanizing regions like Colombo District, Sri Lanka.

This study was conducted from September to March, a period encompassing the Southwest monsoon and subsequent inter-monsoon season in Sri Lanka. These months typically experience higher rainfall and humidity, creating favourable conditions for *Aedes* mosquito breeding. Therefore, this duration selected for the study may be a suitable period to assess the oviposition behavior under peak vector activity influenced by seasonal ecological factors. To minimize the impact of environmental and anthropogenic confounding variables on oviposition behavior, several precautions were taken. Ovitraps were placed in shaded, undisturbed outdoor areas commonly found in residential settings, avoiding locations with high foot traffic or regular cleaning. Households that were selected for the study were informed not use chemical repellents or mosquito control treatments in close proximity to trap locations during the study period. Furthermore, care was taken to select trap sites with minimal presence of alternate breeding containers nearby, or such containers were emptied prior to deployment. While complete elimination of all confounding variables in field settings is not possible, these measures helped ensure that observed oviposition patterns were more directly attributable to the cement types used in the ovitraps.

Among the cement substrates tested, BHC and PCC were associated with significantly lower egg counts and a higher probability of zero oviposition, suggesting that these materials may exert a deterrent effect on *Aedes* mosquitoes. The moderate egg count observed on the OPC substrate, which was higher than that of BHC and PCC, however, lower than the plastic control, likely reflects distinct physicochemical changes imparted to the ovitrap water by the specific chemical composition of OPC. This may be due to the raising of the pH and releasing calcium and other ions into the water due to its mineral content and hydration process, creating an alkaline environment. This altered water chemistry can deter or favor mosquito oviposition depending on species-specific tolerances and preferences. Compared to the plastic control, which is chemically inert and does not alter water quality. This lack of interaction ensures that the water remains stable, with no changes in parameters such as pH, conductivity, or ionic content. Such consistency likely offers a more favorable and predictable environment for mosquito oviposition compared to cement-based substrates that can leach compounds and modify water quality. Consequently, the chemically stable environment of the plastic control ovitrap likely contributed to it receiving the highest number of mosquito eggs in the current study. To ensure the possible repelling effect of mosquitoes for oviposition due to the cement odor, the traps were kept for seven days for seasoning. Therefore, it could be assumed that the potential repelling effect due to cement odor is minimal and attraction is mainly based on the physicochemical properties in the water that are allied with the cement constituents and texture. These findings align with earlier laboratory and semi-field studies that showed physicochemical properties of container surfaces, including surface roughness and leachate chemistry, can influence oviposition site selection by *Aedes* species [34, 35].

Environmental placement also played a critical role. Outdoor ovitraps recorded significantly higher egg counts than indoor traps, reaffirming previous research that highlights outdoor environments as more favorable due to better access, higher humidity, and lower human interference [5]. However, the zero-inflation model also showed that outdoor traps had a higher probability of being egg-negative (i.e., containing no eggs), indicating that when given a choice between outdoor and indoor traps, *Aedes* prefer indoor sites. However, when outdoor traps are chosen, a higher number of eggs tend to be laid in them.

Regarding spatial differences, traps located in Orugodawatta had significantly lower egg counts and higher odds of being egg-negative compared to Kolonnawa. This spatial variation may reflect differences in urban structure, vegetation cover, population density, or microhabitat conditions across Grama Niladhari divisions, as supported by previous studies [29], which found that urban microenvironments significantly affect *Aedes* breeding site productivity.

Among the physicochemical water parameters assessed, only conductivity was found to have a statistically significant negative association with egg counts. This may indicate that higher levels of dissolved ions discourage oviposition. This observation is in consistent with previous findings, that reported reduced egg-laying in response to increased NaCl concentrations [10] However, pH and turbidity were not significantly associated with oviposition, contrasting with some laboratory studies that suggest extremes of pH may reduce egg-laying behavior [36]. The lack of effect here may be due to the narrow pH range in field conditions or behavioral adaptation of *Aedes* to suboptimal water quality.

This distinction is important for vector control strategies, as it implies that the use of less-preferred construction materials may reduce breeding site utilization without necessarily relying on larvicidal or ovicidal interventions. Integrating behavioral insights into urban planning and construction practices could offer a complementary and sustainable approach to dengue prevention, particularly in high-risk, rapidly urbanizing regions.

Future studies should further explore the chemical composition and surface properties of cement types that deter oviposition and assess their long-term effects on mosquito colonization. Additionally, experimental designs incorporating larval survival and adult emergence rates will provide a more comprehensive understanding of the vectorial potential of such breeding sites. While this study was conducted entirely under field conditions to capture natural oviposition behavior, the absence of a controlled laboratory component is acknowledged. In future work, laboratory-based experiments under standardized conditions should be conducted to isolate the effects of cement composition from environmental variables and to validate field observations.

### Limitations

This study, while providing valuable insights into the oviposition behavior of dengue vectors in relation to construction materials, is not without limitations. Firstly, the study was limited to a single Medical Officer of Health (MOH) area in the Colombo District, which may restrict the generalizability of findings to other geographical areas with different climatic, ecological, or socio-demographic profiles. Secondly, species-specific data on oviposition preferences were not separated for *Ae. aegypti* and *Ae. albopictus*, even though both species may have differing behaviors and habitat preferences. Additionally, only three cement types were evaluated, despite a wider variety of cement materials being used in the Sri Lankan construction industry. Further, chemical leachate analysis of each cement type was not conducted, which would have helped to identify the specific components that might act as oviposition deterrents. Temporal variations in weather, rainfall, and mosquito density, which could influence oviposition behavior, were not controlled for across the 7-month data collection period.

## Conclusion

The ovitraps made from Blended Hydraulic Cement (BHC) and Portland Composite Cement (PCC) were significantly less attractive to *Aedes* mosquitoes compared to conventional substrates, suggesting that these materials may possess oviposition-deterrent properties. Additionally, environmental factors such as outdoor placement and water conductivity were shown to significantly affect egg-laying behavior, with outdoor traps attracting more eggs, while higher conductivity levels were associated with reduced oviposition. The results emphasize the critical role of construction environments, particularly in rapidly urbanizing areas, as potential breeding habitats for *Aedes* mosquitoes. These outcomes suggest that construction environments can modify breeding opportunities and influence vector ecology. Understanding how building materials influence mosquito behavior may offer a complementary strategy to vector control, complementing traditional approaches such as chemical interventions and source reduction. However, it is important to note that construction materials are likely to play only a supplementary role in integrated vector management, as the avoidance of natural breeding sites and the behavioral adaptability of *Aedes* remain critical challenges for long-term control. Incorporating environmental modification and material selection into dengue prevention strategies may contribute to cost-effective and sustainable vector control when integrated with existing approaches.

## Supplementary Information

Supplementary Material 1. Figure S1 Goodness of fit of the model (a) The scatter plot of predicted versus observed egg counts (b) The plot of residuals versus the predicted egg counts (c) Binned Residual Plot. Figure S2 Mean egg count (± SE) in ovitraps placed indoors and outdoors across different substrate types. Table S1 Chemical composition of major cement materials used in Sri Lanka. Table S2 Physicochemical properties (mean ± SE and range) of water in ovitraps across different cement substrates. Table S3 Total egg counts recorded for each substrate during the study period. Table S4 Total egg counts recorded in indoor and outdoor ovitraps during the study period.

## Data Availability

Data is provided within the manuscript.

## Abbreviations

GN: Grama niladhari
BHC: Blended hydraulic cement
OPC: Ordinary portland cement
PCC: Portland limestone cement
ZINB: Zero-inflated negative binomial
IVM: Integrated vector management
MOH: Medical officer of health
