# Supplementary material for: Oviposition response of Aedes mosquitoes to different cement types: a field-based study in urban Sri Lanka
Source: BMC Res Notes. 2025 Dec 13;19:29. doi: 10.1186/s13104-025-07610-8 (PMC12817672; doi:10.1186/s13104-025-07610-8)
Supplement: Supplementary file 1 — Supplementary Material 1. Figure S1 Goodness of fit of the model (a) The scatter plot of predicted versus observed egg counts (b) The plot of residuals versus the predicted egg counts (c) Binned Residual Plot. Figure S2 Mean egg count (± SE) in ovitraps placed indoors and outdoors across different substrate types. Table S1 Chemical composition of major cement materials used in Sri Lanka. Table S2 Physicochemical properties (mean ± SE and range) of water in ovitraps across different cement substrates. Table S3 Total egg counts recorded for each substrate during the study period. Table S4 Total egg counts recorded in indoor and outdoor ovitraps during the study period. [file 13104_2025_7610_MOESM1_ESM.docx]

**Supplementary Materials**


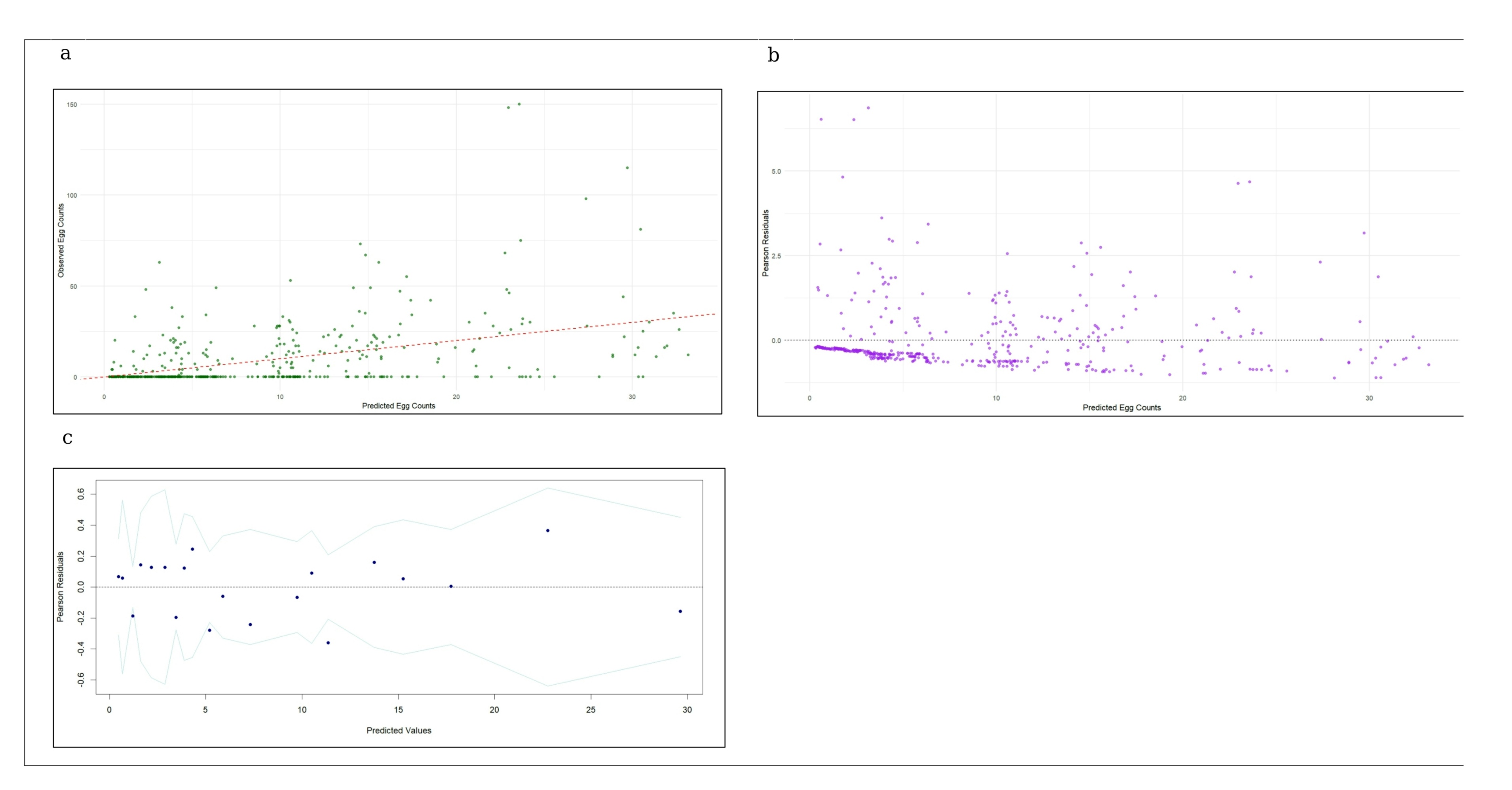


**Figure S1** Goodness of fit of the model (a) The scatter plot of predicted versus observed egg counts (b) The plot of residuals versus the predicted egg counts (c) Binned Residual Plot


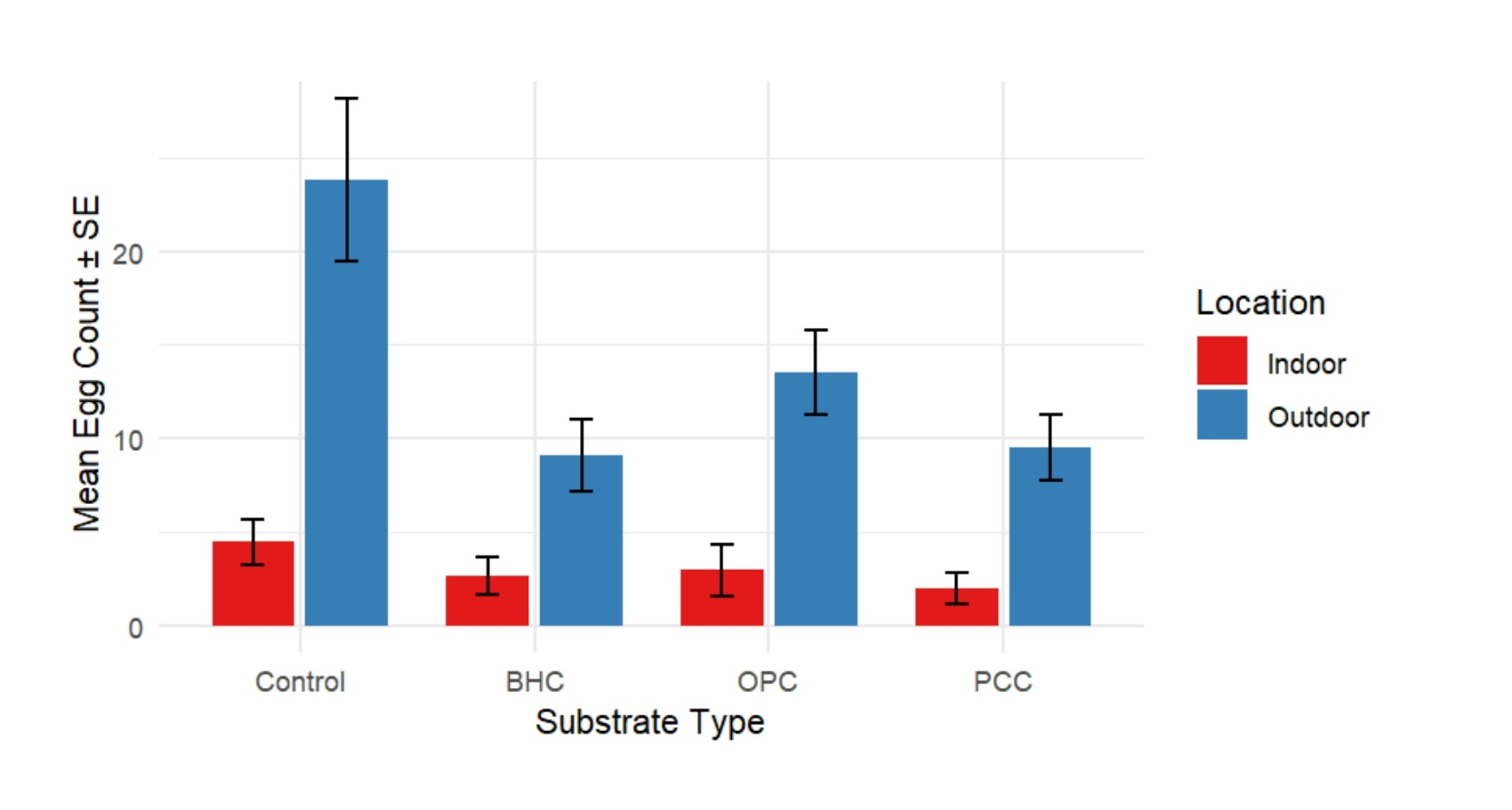


**Figure S2** Mean egg count (± SE) in ovitraps placed indoors and outdoors across different substrate types.

**Table S1** Chemical composition of major cement materials used in Sri Lanka

| Type of Cement  Chemical Compound | Ordinary Portland Cement % | Portland Composite Cement % | Blended Hydraulic Cement % |
| --- | --- | --- | --- |
| CaO | 53.6 | 55.05 | 47 |
| SiO_2_ | 28.7 | 25.88 | 23.5 |
| Al_2_O_3_ | 13.5 | 3.77 | 12.9 |
| MgO | 2.21 | 5.06 | 1.74 |
| Fe_2_O_3_ | 2.27 | 3.01 | 2.04 |
| SO_3_ | 2.9 | 2.72 | 2.21 |
| Loss on ignition | 2.05 | 3.72 | 1.05 |

**Table S2** Physicochemical properties (mean ± SE and range) of water in ovitraps across different cement substrates

| **Substrate** | **pH** | | **Conductivity (µS/cm)** | | **Turbidity (NTU)** | |
| --- | --- | --- | --- | --- | --- | --- |
|  | Mean | Range | Mean | Range | Mean | Range |
| Control | 10.68 ± 1.18 | 7.70–13.70 | 109.83 ± 37.30 | 9.00–237.00 | 1.32 ± 3.46 | 0.00–34.72 |
| BHC | 11.21 ± 1.18 | 8.70–13.80 | 160.54 ± 129.18 | 12.10–1131.00 | 2.15 ± 4.25 | 0.00–31.01 |
| OPC | 11.10 ± 1.07 | 9.20–13.40 | 173.09 ± 72.60 | 47.00–434.00 | 2.12 ± 4.64 | 0.00–38.81 |
| PCC | 10.93 ± 1.15 | 8.20–13.60 | 136.86 ± 68.01 | 27.00–636.00 | 1.86 ± 2.92 | 0.00–19.40 |

**Table S3** Total egg counts recorded for each substrate during the study period

| **Substrate** | **Total Eggs** |
| --- | --- |
| Control | 1698 |
| BHC | 708 |
| OPC | 994 |
| PCC | 693 |

**Table S4** Total egg counts recorded in indoor and outdoor ovitraps during the study period

| **Indoor/Outdoor** | **Total Eggs** |
| --- | --- |
| Indoor | 734 |
| Outdoor | 3359 |
